# Effect of Probiotics and Prebiotics on Immune Response to Influenza Vaccination in Adults: A Systematic Review and Meta-Analysis of Randomized Controlled Trials

**DOI:** 10.3390/nu9111175

**Published:** 2017-10-27

**Authors:** Wei-Te Lei, Pei-Ching Shih, Shu-Jung Liu, Chien-Yu Lin, Tzu-Lin Yeh

**Affiliations:** 1 Department of Pediatrics, Hsinchu MacKay Memorial Hospital, Hsinchu 30071, Taiwan; Weite.lei@gmail.com (W.-T.L.); mmhped.lin@gmail.com (C.-Y.L.); 2 Department of Family Medicine, Hsinchu MacKay Memorial Hospital, Hsinchu 30071, Taiwan; 2053.2053@mmh.org.tw; 3 Departmental of Medical Library, MacKay Memorial Hospital, Tamsui Branch, New Taipei City 25160, Taiwan; sjliu@mmh.org.tw

**Keywords:** probiotics, prebiotics, seroprotection, seroconversion, influenza vaccine, systematic review, meta-analysis

## Abstract

We conducted a meta-analysis to evaluate the effects of probiotics and prebiotics on the immune response to influenza vaccination in adults. We conducted a literature search of Pubmed, Embase, the Cochrane Library, the Cumulative Index to Nursing and Allied Health (CINAHL), Airiti Library, and PerioPath Index to Taiwan Periodical Literature in Taiwan. Databases were searched from inception to July 2017. We used the Cochrane Review risk of bias assessment tool to assess randomized controlled trial (RCT) quality. A total of 20 RCTs comprising 1979 adults were included in our systematic review. Nine RCTs including 623 participants had sufficient data to be pooled in a meta-analysis. Participants who took probiotics or prebiotics showed significant improvements in the H1N1 strain seroprotection rate (with an odds ratio (OR) of 1.83 and a 95% confidence interval (CI) of 1.19–2.82, *p* = 0.006, *I*^2^ = 0%), the H3N2 strain seroprotection rate (OR = 2.85, 95% CI = 1.59–5.10, *p* < 0.001, *I*^2^ = 0%), and the B strain seroconversion rate (OR = 2.11, 95% CI = 1.38–3.21, *p* < 0.001, *I*^2^ = 0%). This meta-analysis suggested that probiotics and prebiotics are effective in elevating immunogenicity by influencing seroconversion and seroprotection rates in adults inoculated with influenza vaccines.

## 1. Introduction

Influenza is an acute viral respiratory infection caused by RNA viruses and results in fever and myalgia in infected people. Although influenza is self-limited in most cases, it can cause serious diseases such as pneumonia, myocarditis, and encephalitis, which result in high morbidity and significant mortality in children, pregnant women, and the elderly. In general, epidemically seasonal influenza leads to three to five million severe illness cases and around 250,000 to 500,000 deaths in the world yearly. Even more, influenza pandemics are unpredictable and can have significant impacts on human health and the economy worldwide. Currently, annual influenza vaccines are the main intervention for minimizing both the mortality and morbidity of influenza [1].

Although vaccination in children, adolescents, and young adults can help prevent influenza infection by 70% to 90%, among people older than 65 years old its protective effects only range from 30% to 40%, according to a previous report [2]. Aging is accompanied by a decline in both innate and adaptive immune responses. Suboptimal cytotoxicity of natural killer (NK) cells, phagocytosis, B cell antibody production, and T cell cellular immune response result in poorer responses to both infection and immunization [3,4]. This immunosenescence caused by aging limits the protective effects of vaccination in older adults. Adjuvants such as heat-labile enterotoxin have been co-administrated with the inactivated vaccine to improve potency. However, there remain some safety concerns regarding this process [5].

Probiotics such as *Lactobacillus* and *Bifidobacterium* are live bacteria that are beneficial to the host when administrated in proper amounts [6]. The use of probiotics has been shown to not only modulate both innate and adaptive immunity in the elderly, but also reduce the length of infection in children and adults [7,8,9]. Prebiotics like oligosaccharides are substances that stimulate the metabolism and growth of commensal enteric bacteria that benefit the host. It has been proven that prebiotics can modulate B cell response and augment the Th1-dependent immune response [10,11,12]. Both probiotics and prebiotics have been shown in clinical trials to have protective effects against influenza infection. In addition, there have been studies focused on the usefulness of adjuvant supplementation of probiotics or prebiotics with measles vaccination. From this point of view, probiotic or prebiotic supplementation appears to be an attractive and safe way to enhance the effectiveness of influenza vaccines.

Several randomized controlled trials (RCTs) have evaluated the influence of probiotic or prebiotic consumption on individual immune responses induced by an influenza vaccine, but no systematic review has examined the link between the consumption of probiotics or prebiotics and immunogenicity outcomes in adults vaccinated with an influenza vaccine. Furthermore, results of former studies concerning the efficacy of supplementation in relation to subsequent serum antibody changes after influenza vaccination remain inconclusive. The present systematic review and meta-analysis thus aim to explore the effectiveness of probiotics and prebiotics on immune functions in adults inoculated with an influenza vaccine.

## 2. Methods

This systematic review and meta-analysis were conducted in accordance with the Preferred Reporting Items for Systematic review and Meta-Analysis Protocols (PRISMA-P) guidelines [13] (Table S1).

We searched the following databases from inception to the end of July 2017: Embase, PubMed, the Cochrane Library, the Cumulative Index to Nursing and Allied Health (CINAHL), the Airiti Library, and the PerioPath Index to the Taiwan Periodical Literature in Taiwan.

We used the keywords “influenza vaccine” AND “probiotics” OR “prebiotics” OR “synbiotics” in our search. Our strategy is shown in Table S2. To ensure a comprehensive search, we did not limit the language, year, or type of publication. Two authors (PCS and SJL) conducted the search independently, and disagreements were resolved through discussion with the third author (WTL).

### 2.1. Study Selection and Methodological Quality Assessment

After the initial search, two independent reviewers (PCS and TLY) assessed each publication to determine whether the article met the inclusion criteria for systematic review and meta-analysis. The RCTs included met all of the following eligibility criteria: (1) focused on human adults; (2) includes a controlled group in the study design; (3) includes inoculation of an influenza vaccine and use of probiotics, prebiotics, or synbiotics by the intervention group; and (4) reports at least one immunological response to vaccination. We excluded the following: (1) articles irrelevant to the topic, (2) duplicate publications, (3) trials of a cross-over study design, and (4) studies in which the control arm received an effective intervention rather than a placebo.

Quality assessment of all included studies was conducted independently by two researchers (WTL and TLY) using the Cochrane Review risk of bias assessment tool [14]. The adequacy of randomization, allocation concealment, blinding methods, implementation of the intention-to-treat analysis, dropout rate, complete outcome data, selective data reporting, and other biases were assessed. Each domain was categorized as low, high, or unclear.

### 2.2. Data Extraction and Analysis

Three authors (T.L.Y., C.Y.L., and W.T.L.) independently extracted the data from all included studies, and the following data were collected: first author’s name, year of publication, country of publications, number of patients, age of patients, sex ratio of patients, type of intervention, type of vaccine, clinical outcome measures, and severe adverse effects. To evaluate influenza vaccine immunogenicity, factors affecting antibody Geometric Mean Titer (GMT) and seroprotection and seroconversion rates were extracted from the trials. Such factors included Hemagglutination inhibition (HI) antibody titers, serum immunoglobulins, cytokine secretion, lymphocyte proliferation, immune cell phenotypes, compliance variables, biochemical markers, and episode or duration of upper respiratory tract infection or flu-like illness. Our objective was to determine the influence of probiotics and prebiotics on the seroprotection and seroconversion rates of adults after influenza vaccination. HI antibody titer equals the maximum dilution capable of inhibiting the agglutination of guinea pig red blood cells with the influenza viruses under standardized conditions [15]. Seroconversion rate is defined as the proportion of volunteers achieving at least a fourfold increase in antibody titer after vaccination. Seroprotection rate is defined as the proportion of volunteers achieving an influenza antibody titer greater than or equal to 40 in an HI test [16].

The European Committee for Proprietary Medicinal Products (CPMP) guidelines [17] set the cut-off levels of vaccine immunogenicity for a population over the age of 60 years as at least a 60% seroprotection rate, at least a 30% seroconversion rate, and an over 2.5-fold increase in antibody GMT. Each of the vaccine antigens must meet at least one of the above criteria in the CPMP guidelines.

Meta-analysis was conducted when the trials had acceptable clinical homogeneity and statistical heterogeneity. Due to the significant heterogeneity expected among the studies, a random effects model was employed using DerSimonian and Laird’s method [18,19]. To evaluate the differences in immunogenicity between the intervention and the control groups, dichotomous data were analyzed using an odds ratio (OR) with 95% confidence intervals (CI). Heterogeneity was quantified using the Cochran Q TEST and *I*^2^ statistics [19]. Potential publication bias was assessed by observing the symmetry of funnel plots and by using Egger’s test [20]. Meta-analysis was performed using Review Manager (RevMan) [Computer program]. Version 5.3. Copenhagen: The Nordic Cochrane Centre, The Cochrane Collaboration, 2014. Comprehensive Meta-Analysis version 3 (Biostat, Englewood, NJ, USA) was used to conduct Egger’s test and the meta-regression.

## 3. Results

### 3.1. Description of Studies and Quality Assessment

**Figure 1:**
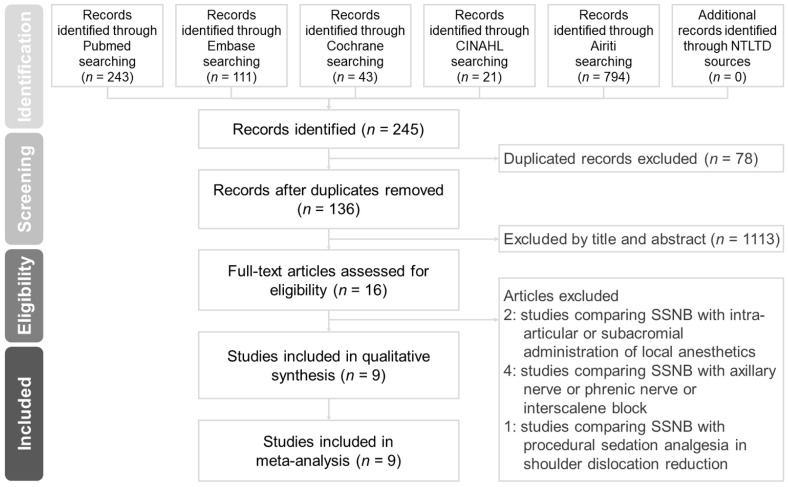
Preferred Reporting Items for Systematic review and Meta-Analysis (PRISMA) flow diagram.

Figure 1 shows the search process and outcomes. A total of 19 publications with 20 RCTs were included for our systematic review [21,22,23,24,25,26,27,28,29,30,31,32,33,34,35,36,37,38,39]. Two trials (a pilot and a confirmatory study) with different patient numbers, treatment protocols, and years of study were published together [35]. Thirteen trials focused on probiotics [22,26,27,28,29,30,31,32,33,34,35,36], while the other six RCTs focused on prebiotics [23,24,25,37,38,39]. Only one study concentrated on synbiotics [21]. Akatsu et al. published a letter to the editor [28] and an original article [27] in the same year. As the study methods were different, we included both of the publications in our review.

Most of the included studies had low bias, as shown by our quality assessment using the Cochrane assessment tool. The detailed quality assessment of each included study is shown in Table S3.

### 3.2. Demographics

The characteristics of the included trials are shown in Table 1. These studies were conducted worldwide, with six trials in Japan [22,23,24,27,28,33], three trials in the USA [32,37,38], two studies each in Spain [31,36] and the UK [21,25], one publication in France [35], and one trial each in Australia [34], Belgium [29], Italy [30], Germany and Denmark [26], and Chile [39]. Seven RCTs enrolled healthy adults or older adults [21,25,26,30,32,34,36], and two trials enrolled healthy older adults [38,39]. In another eleven trials, subjects living in hospitals, nursing homes, or long-term care facilities were enrolled [22,23,24,27,28,29,31,33,35,37]. Participants fed by enteral tube or percutaneous endoscopic gastrostomy were enrolled in three studies conducted in Japan [23,24,27].

A total of 1979 participants with an average age of 58.1 years were enrolled. The male to female ratio was 2.2.

### 3.3. Intervention

Ten RCTs used *Lactobacillus* [22,26,28,29,30,31,32,34,35,36] as a probiotic. Four studies [21,27,30,33] selected *Bifidobacterium*. *Lactobacillus casei or paracasei* were the most commonly used probiotics in the included studies [22,26,28,29,30,35], followed by *Lactobacillus fermentum* [34,36], *Lactobacillus rhamnosus GG* [32], and *Lactobacillus plantarum* [31]. One study compared two different probiotics, *Bifidobacterium animalis ssp. lactis* and *Lactobacillus paracasei subsp. paracasei* [30]. Another trial compared the effect of *Lactobacillus plantarum* in different doses [31].

Prebiotics were supplied in different combinations across the included studies. Fructo-oligosaccharide was the most commonly used prebiotic component [37,38,39] mixed with different oils [38], triglycerols, vitamins, or minerals [37,38], followed by galacto-oligosaccharides [23,24] mixed with a bifidogenic growth stimulator or fermented milk products. One trial selected long-chain inulin and oligofructose [25].

The supplementation duration ranged from 2 to 28 weeks, with an average of 7, 16, and 8 weeks in probiotics, prebiotics, and synbiotics, respectively. Out of 1979 total participants, 49 individuals had severe adverse effects.

Almost all of the included studies used a trivalent inactivated influenza vaccine. Only two RCTs selected a live attenuated influenza vaccine [23,32]. In one trial, all participants were both vaccinated with a trivalent inactivated influenza vaccine and the pneumococcal polysaccharide vaccine 23 [39].

### 3.4. Outcome Measurement

We excluded one RCT [27] from our meta-analysis when considering seroprotection rate to prevent a possible overestimation of the real effect from the results. The excluded study had reported data in which the HI was higher than 20, which is lower than the amount required by the definition of seroprotection. After performing a thorough review of an RCT conducted in 2015 [26], we found that the numbers were not compatible with the data in the article. As we had reasonable doubts concerning the accuracy of the numbers in the article, we excluded the article from our meta-analysis.

### 3.5. Efficacy of Probiotics and Prebiotics in Participants Inoculated with an Influenza Vaccine Compared with Controls

Seven RCTs [23,24,25,32,33,35,37] including 389 participants had sufficient data to be pooled for an analysis of seroprotection rate. Meanwhile, a total of six RCTs [25,28,32,35,37] including 553 participants were enrolled for our meta-analysis to determine seroconversion rate. The average age of all participants was 74.8 years old. The seroprotection rates in those who took probiotics or prebiotics with the H1N1, H3N2, and influenza B vaccines were 53%, 84%, and 53%, respectively. The overall seroconversion rates for the H1N1, H3N2, and influenza B vaccines were 37%, 65%, and 50%, respectively.

**Figure 2:**
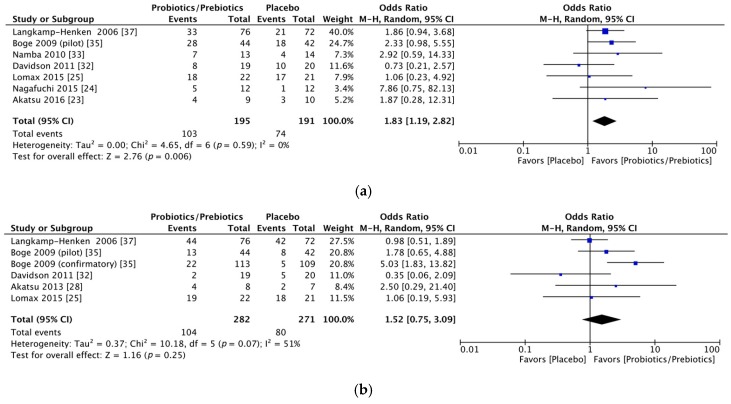
Forest plots of seroprotection and seroconversion rate of H1N1 strain. (**a**) Forest plot of seroprotection rate of H1N1 strain; (**b**) Forest plot of seroconversion rate of H1N1 strain. The bold data represents total participants of all included studies and the Odds ratio (OR) between the probiotics/prebiotics group and the placebo group. The diamond stands for the pooled OR. Weights are from random-effects model. CI: confidence interval.

**Figure 3:**
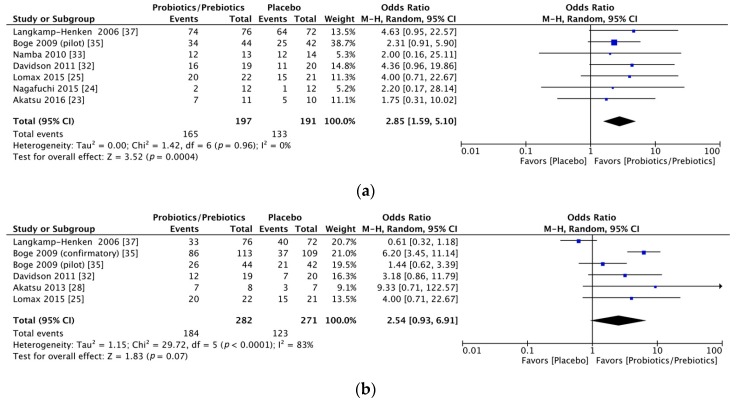
Forest plots of seroprotection and seroconversion rate of H3N2 strain. (**a**) Forest plot of seroprotection rate of H3N2 strain; (**b**) Forest plot of seroconversion rate of H3N2 strain. The bold data represents total participants of all included studies and the Odds ratio (OR) between the probiotics/prebiotics group and the placebo group. The diamond stands for the pooled OR. Weights are from random-effects model. CI: confidence interval.

**Figure 4:**
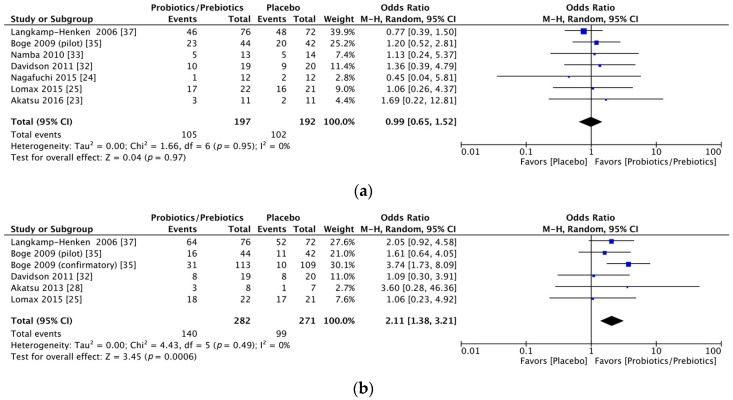
Forest plots of seroprotection and seroconversion rate of B strain. (**a**) Forest plot of seroprotection rate of B strain; (**b**) Forest plot of seroconversion rate of B strain. The bold data represents total participants of all included studies and the Odds ratio (OR) between the probiotics/prebiotics group and the placebo group. The diamond stands for the pooled OR. Weights are from random-effects model. CI: confidence interval.

Significant immunogenicity differences were documented between those who took probiotics or prebiotics and the controls. For the H1N1 vaccine, the OR for seroprotection was 1.83, with a 95% CI of 1.19–2.82, *I*^2^ = 0%, *p* = 0.006 (Figure 2a), whereas the OR for seroconversion was 1.52, with a 95% CI of 0.75–3.09, *I*^2^ = 51%, *p* = 0.25 (Figure 2b). With regards to the H3N2 vaccine, there was a significant difference in the seroprotection rate (probiotics/prebiotics vs. controls, OR = 2.85, 95% CI = 1.59–5.10, *I*^2^ = 0%, *p* < 0.001) (Figure 3a) but not the seroconversion rate (OR = 2.54, 95% CI = 0.93–6.91, *I*^2^ = 83%, *p* = 0.07) (Figure 3b). Furthermore, for the influenza B vaccine, a significant difference was noted in the seroconversion rate (OR = 2.11, 95% CI = 1.38–3.21, *I*^2^ = 0%, *p* < 0.001) (Figure 4b) and not the seroprotection rate (OR = 0.99, 95% CI = 0.65–1.52, *I*^2^ = 0%, *p* = 0.97) (Figure 4a).

### 3.6. Subgroup Meta-Analysis of Influenza Vaccine Immunogenicity in Participants Supplied with Different Supplements

**Figure 5:**
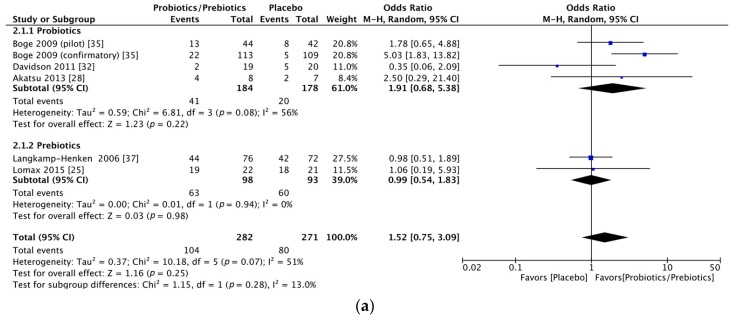

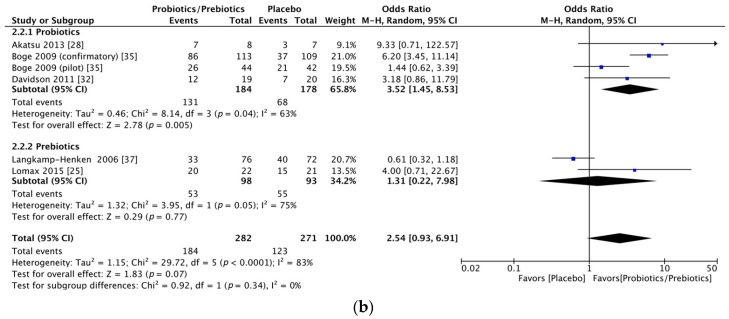
Forest plots of subgroup analysis by intervention type of seroconversion rate of H1N1 and H3N2 strains. (**a**) Forest plot of subgroup analysis by intervention type of seroconversion rate for influenza H1N1 strain; (**b**) Forest plot of subgroup analysis by intervention type of seroconversion rate for influenza H3N2 strain. The bold data represents total participants of all included studies and the Odds ratio (OR) between the probiotics/prebiotics group and the placebo group. The diamond stands for the pooled OR. Weights are from random-effects model. CI: confidence interval.

Due to the relatively moderate heterogeneity of seroconversion rates, we performed a subgroup analysis according to the intervention of probiotics or prebiotics. In the H1N1 seroconversion rate, the results remained unchanged except for a decrease in heterogeneity (OR = 1.91, 95% CI = 0.68–5.38, *I*^2^ = 56%, *p* = 0.22; OR = 0.99, 95% CI = 0.54–1.83, *I*^2^ = 0%, *p* = 0.98, forest plot in Figure 5a) after dividing all of the participants into probiotic and prebiotic groups. For the H3N2 seroconversion rate, the favorable effect was shown in the probiotics group (OR = 3.52, 95% CI = 1.45–8.53, *I*^2^ = 63%, *p* = 0.005) but not in the prebiotics group (OR = 1.31, 95% CI = 0.22–7.98, *I*^2^ = 75%, *p* = 0.77, forest plot in Figure 5b).

### 3.7. Subgroup Meta-Analysis of the Immunogenicity of the Influenza Vaccine in Participants Divided into Different Health Statuses

**Figure 6:**
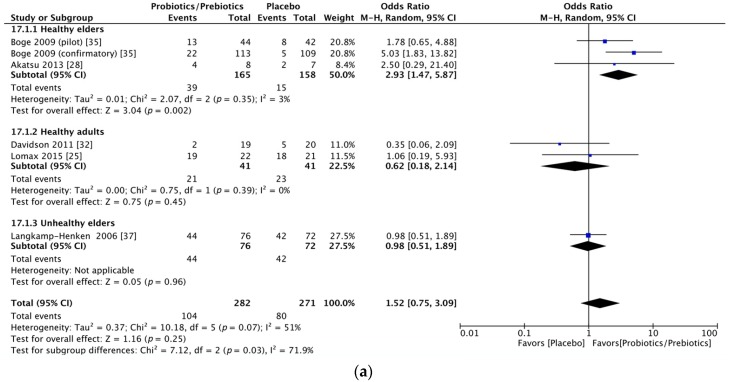

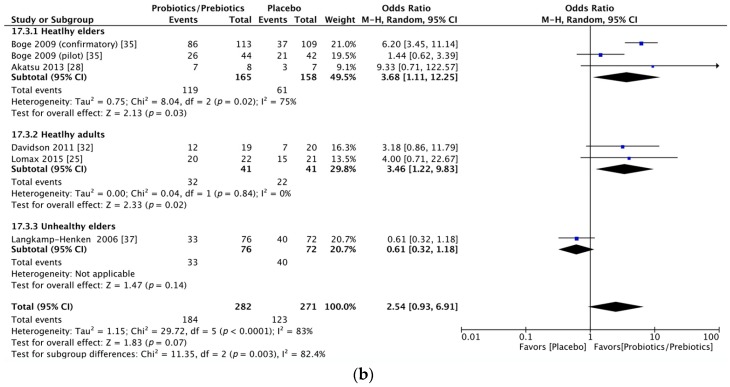
Forest plots of subgroup analysis by participants of seroconversion rate of H1N1 and H3N2 strains. (**a**) Forest plot of subgroup analysis by health status of participants of seroconversion rate for influenza H1N1 strain; (**b**) Forest plot of subgroup analysis by health status of participants of seroconversion rate for influenza H3N2 strain. The bold data represents total participants of all included studies and the Odds ratio (OR) between the probiotics/prebiotics group and the placebo group. The diamond stands for the pooled OR. Weights are from random-effects model. CI: confidence interval.

Given the persistent heterogeneity, we performed another subgroup meta-analysis based on the different health statuses of the participants. We found that participants in the included studies could be grouped into the following three categories: healthy young to middle-aged adults, healthy older adults, and frail or hospitalized older adults. The heterogeneity of the seroconversion rate was lowered as a result of the subgroup meta-analysis (Figure 6a,b). In addition, among the two indexes (seroprotection and seroconversion rates) used to evaluate the effects of probiotics and prebiotics in relation to the three strains of influenza vaccine, participants from the healthy older adult category had the best response to influenza vaccination followed by the healthy young to middle-aged adults and then the frail or hospitalized older adults (Table 2).

### 3.8. Meta-Regression

To examine the heterogeneity of the current analysis, a meta-regression analysis was also done using the age of participants and the duration of supplementation as moderators in the single meta-regression. We found that the effect of probiotics or prebiotics on immune responses to all of the influenza vaccine strains was not significantly confounded by age. The effects of probiotics and prebiotics on the seroconversion rate against the influenza B strain (slope = 0.14, *p* = 0.049) and the seroconversion rate against the influenza H1N1 strain (slope = 0.21, *p* = 0.043) were significantly confounded by the duration of supplementation.

### 3.9. Assessment of Publication Bias

Our funnel plots are symmetric upon inspection (Figures S1 to S6). Egger’s regression confirmed that there was no statistically significant publication bias with a *p* value > 0.05 (Table S4).

## 4. Discussion

To the best of our knowledge, this is the first systemic review and meta-analysis to be conducted on the effect of supplementary probiotic and prebiotic use on influenza vaccine efficacy in adults. In our analysis, we found that the supplementation of influenza vaccines with probiotics or prebiotics before vaccination increased the immunogenicity to specific influenza viral strains, including the H1N1, H3N2, and B strains. The current study included seven RCTs related to seroprotection rates that revealed a significantly better protective effect in those who took probiotics or prebiotics orally as an adjuvant for the parenterally administered H1N1 and H3N2 vaccines. In addition, pooled results from the six studies focused on seroconversion rate showed a significantly enhanced efficacy of the influenza B vaccine in those who consumed probiotics or prebiotics.

In our analysis, the participants supplemented with probiotics or prebiotics not only satisfied at least one of the CPMP guidelines [17] for all influenza strains (seroprotection rate against H3N2 and seroconversion rates against the H1N1, H3N2, and B strains), but also displayed higher seroprotection and seroconversion rates against the H1N1, H3N2, and B strains than those of the control group. For one RCT [37] included in our analysis, we might have underestimated the seroprotection rate against H1N1, as an HI equal to or above 100 was used as the standard. In addition, we excluded another study that defined seroprotection as HI of 20 or over [27]. We have more confidence in our results because our choice to underestimate rather than overestimate the real effects led to solid results on the benefits of probiotics and prebiotics.

Previous RCTs on the efficacy of the use of probiotics and prebiotics as supplements for amplifying the effect of influenza vaccines have reported inconsistent conclusions and a lack of evidence to support such a use of probiotics or prebiotics in clinical practice. Our results are consistent with the majority of the 20 enrolled RCT studies; only three trials showed results that were inconsistent with ours [29,36,39]. The inconsistency might be attributed to not only study design, such as the type and duration of supplementation, but also the demographic characteristics of the participants, more specifically age and health status. We tried to investigate the possible confounding effect of these variables on the probiotic or prebiotic efficacy in relation to an influenza vaccine. We found that the duration of supplementation, and not the age of participants, had a significant impact on the participants’ response to probiotics or prebiotics. Longer duration of supplementation rendered participants more sensitive to vaccine stimulation. In previous reports [29,40], aging has been suggested as the reason for a poorer immune response to both influenza vaccines and probiotic stimulation. However, younger ages may not show positive effects from probiotic or prebiotic supplementation because this age group has higher possibility of an optimal response to vaccination. In our study, we found that health status plays a more important role than age. Our analysis showed that healthy older adults obtained the most benefit from probiotics and prebiotics, compared with the other two types of participants. The solid evidence from our results has clinical importance: clinicians can use our results to make tailored suggestions for specific populations to augment vaccine immunogenicity.

Compliance may also be a confounding factor in interventional studies. In current analysis, twelve studies recorded the compliance. Three studies further confirmed intake of probiotics via culture-based mechanism, using qPCR, counting fecal bacteria numbers, or detecting fecal probiotics strains (Table S5). However, only 4 of the 12 studies were included in the meta-analysis with only 1 study declaring not good compliance. We found that compliance had no impact on the current results. Moreover, the strains of probiotics may also play a vital role. In the further subgroup analysis based on different probiotics strains, we found that non-LGG strains (i.e. *L. casei, L. paracasei*, and *B. longum*) had positive effects on immunogenicity changes in all vaccine strains. However, LGG showed no effects in any of the three vaccine strains (Table S6). Further studies are required to clarify the influences of different probiotics strains.

The underlying mechanisms of probiotics and prebiotics in terms of their effect on immune functions may differ. Probiotics induce cellular immunity in phagocyte and NK cells [33,41] and promote IgA secretion into saliva to enhance the vaccine effects [35,42]. Furthermore, the metabolites of probiotics, such as short-chain fatty acids, and the peptidoglycan components of probiotics appear benefits on both the host gut epithelium and microbiota by modulating the immune function [43,44]. It has also been shown that probiotics shorten the duration and decrease incidence of infections in the elderly during winter [33]. Prebiotics promote the development of the bifido flora in the intestines and enhance both the production of interferon γ and NK cell activity [45,46,47]. In addition, interferon γ is produced by Th1 cells and has a protective role against influenza infection through its antiviral effects.

Prebiotics are generally considered to promote the viability or the function of probiotics by their fermentation. However, no previous studies have directly compared the efficacy of prebiotics with that of probiotics in improving the immune response to an influenza vaccine. In our analysis, although the comparison was not direct, the subgroup analysis disclosed that supplementation with probiotics achieved more immunogenicity changes than supplementation with prebiotics (Table 3). Nagafuchi et al. further showed that the seroprotective effect was maintained for a longer period when fermented milk (probiotic) was given with a bifidogenic growth stimulator and galacto-oligosaccharide (prebiotic) in enterally-fed older adults vaccinated with H1N1 [24]. Therefore, a simultaneous supply of prebiotics and probiotics might be an effective method of enhancing immune reactions to an influenza vaccine.

A strength of the current study is the low heterogeneity of the pooled analysis. Furthermore, the trials included in our analysis were collected from numerous databases and comprised studies in different languages drawing from different perspectives and cultures.

There are several limitations to the present meta-analysis. First, the outcomes were the rates of seroprotection and seroconversion, not the changes in antibody geometric mean titer (GMT) due to influenza vaccination. The main reason for this was that only a few of the included studies recorded the antibody titers before and after vaccination. Second, there was only one trial with a subgroup analysis of synbiotics and no trials investigating probiotics or prebiotics versus synbiotics, thereby limiting the comparison of different supplements. Third, due to the limited number of included studies and thus insufficient data on basic immune status and original antibody titers against influenza, it was not possible to perform more subgroup analyses or meta-regressions to examine the impact of variables that may influence the heterogeneity of some observed results in our study. Finally, the medications used by the hospitalized patients in the studies might have been confounding factors, and thus require further clarification; however, none of the included studies provided data on medication records.

## 5. Conclusions

The present meta-analysis revealed that both prebiotics and probiotics can enhance the immunogenicity of a seasonal influenza vaccine in terms of the seroconversion and seroprotection rates in adults, especially in healthy older adults. Longer durations of supplementation had a linear effect on vaccine stimulation. We suggest that either prebiotics or probiotics can be used in adults, especially healthy older adults, prior to seasonal influenza vaccination. Further large RCTs focusing on the optimal dose, duration, and the synergic effect of a combination of probiotics and prebiotics are required to validate these findings.

## Figures and Tables

**Table 1:** Characteristics of randomized clinical trials using probiotics/prebiotics/synbiotics on Influenza-vaccinated adults.

Reference	Country (Tx Duration)	Population (M%:F%)	Age Mean	Intervention:Control	Intervention	Type of Vaccine	Outcome Measure	Severe AEs
Olivares 2007 [36]	Spain (4 weeks)	50 healthy adults (62%:38%)	33.00	25:25	*L. fermentum* CECT5716 1 × 10^10^ CFU daily	H1N1: New Caledonia/20/99 H3N2: A/Fujian/411/2002 B: Shanghai/361/2002	Total plasma Ig/cytokine concentration/lymphocyte subpopulation/pattern of subsequent illness	Nil
French & Penny 2009 [34]	Australia (6 weeks)	47 healthy adults (41%:59%)	31.55	15:32	*L. fermentum* VRI 003 1 × 10^9^ CFU	H1N1: New Caledonia/20/99 H3N2: California/7/2004 B: Malaysia/2506/2004	HI titers/titers to Measles, Varicella zoster antigens/patterns of subsequent Illness	NR
Boge 2009 [35] (pilot)	France (7 weeks)	68 healthy adults in nursing home (44%:56%)	83.64	44:42	*L. casei* DN-114 001 twice daily	H1N1: New Caledonia/20/99 H3N2: Wisconsin/67/2005 B: Malaysia2506/2004	HI titers/seroconversion rate/seroprotection rate	10
Boge 2009 [35] (confirmed)	France (13 weeks)	222 elders in nursing home (33%:67%)	84.64	113:109	*L. casei* DN-114 001 twice daily	H1N1: New Caledonia/20/99 H3N2: California/7/2004 B: Shanghai/361/2002a B: Jiangsu/10/2003a	HI titers/seroconversion rate/seroprotection rate	30
Namba 2010 [33]	Japan (2 weeks)	27 healthy elders in health care facility (11%:89%)	86.70	13:14	*B. longum* BB536 1 × 10^11^ CFU daily	H1N1: New Caledonia/20/99 H3N2: Wyoming/3/2003 B: Shanghai/361/2002	HI titers/NK cell activity, neutrophil bactericidal and phagocytic activity/cell-mediated immunity/pattern of subsequent illness	NR
Davidson 2011 [32]	USA (4 weeks)	42 healthy adults (38%:62%)	33.30	21:21	*L. GG* 1 × 10^10^ CFU twice daily	H1N1: Solomon Islands/3/2006 H3N2: Wisconsin/67/2005 B: Malaysia/2506/2004	Hi titers/seroconversion rate	1
Van Puyenboreck 2012 [29]	Belgium (3 weeks)	737 healthy adults in nursing home (25%:75%)	84.06	375:362	*L. casei Shirota* 6.5 × 10^9^ CFU twice daily	H1N1: Solomon Islands /3/2006 IVR-145 H3N2: Wisconsin /67/2005 B: Malaysia /2506/2004	HI titers/seroconversion rate/seroprotection rates/pattern of subsequent illness	NR
Rizzardini 2012 [30]	Italy (6 weeks)	211 healthy adults (44%:56%)	33.20	109:102	BB-12^®^ (DSM15954) 1 × 10^9^ CFU *L. casei* 431^®^ (ATCC55544) daily	H1N1: Brisbane/59/2007 H3N2: Uruguay/716/2007 B: Florida/4/2006	Total plasma Ig/vaccine-specific Ig/salivary Ig/total salivary Ig/plasma interferon-γ, IL-2, IL-10/NK cell activity/CD4+T-lymphocytes/phagocytosis	Nil
Bosch 2012 [31]	Spain (12 weeks)	60 adults in nursing home (NR)	65–85	19:14:15 Group A: 19 Group B: 14 Control: 15	*L. plantarum* CECT7315/7316 daily Group A: 5 × 10^9^ CFU Group B: 5 × 10^8^ CFU	H1N1: Solomon Islands/3/2006 H3N2: Wisconsin/67/2005 B: Malaysia/2506/2004	HI titers/total plasma Ig/Influenza-specific Ig/pattern of subsequent illness/fecal Microbiota	NR
Akatsu 2013a [28] (letter)	Japan (12 weeks)	15 healthy adults in nursing home (47%:53%)	75.74	8:7	*L. paracasei* MoLac 1 × 10^11^ CFU	H1N1: Brisbane/59/2007 H3N2: Uruguay/716/2007 B: Brisbane/60/2008	HI titers/total serum Ig/seroconversion rate/NK cell activity and neutrophil phagocytic activity	NR
Akatsu 2013b [27] (paper)	Japan (12 weeks)	45 enteral tube feeding hospitalized adults (29%:71%)	81.70	23:22	Bifidobacterium strain, BB536 5 × 10^10^ CFU twice daily	H1N1: Brisbane/59/2007 H3N2: Uruguay/716/2007 B: Brisbane/60/2008	HI titers/total plasma Ig/NK cell activity/innate immunity	Nil
Jespersen 2015 [26]	German, Denmark (6 weeks)	1104 healthy adults (41%:59%)	31.45	553:551	*L. casei* 431 (ATCC55544) 1 × 10^9^ CFU daily	H1N1: California/7/2009 H3N2: Perth/16/2009 B: Brisbane/60/2008	HI titers/influenza A-specific antibodies/seroconversion rate/pattern of subsequent illness	5
Maruyama 2016 [22]	Japan (6 weeks)	42 elders in nursing home (19%:81%)	87.15	21:21	*L. paracasei* MCC 1849 1 × 10^11^ CFU daily	H1N1: California/7/2009 pdm09 H3N2: Texas/50/2012 B: Massachusetts/2/2012 (Yamagata lineage)	HI titers/total plasma Ig/NK cell activity, neutrophil phagocytic and bactericidal activity/pattern of subsequent illness:	Nil
Enani 2017 [21]	UK (8 weeks)	112 healthy adults (NR)	18–35 60–85	Young group: 31:31 Older group: 29:33	*B. longum* 10^9^ CFU/day with GI-OS 8 g/day	H1N1: California/7/2009 H3N2: Perth/16/2009 B: Brisbane/60/2008	B/T cell phenotyping/re-stimulation of PBMC/anti-CMV IgG Ab	NR
Bunout 2002 [39]	Chile (28 weeks)	66 healthy elders (NR, but similar %)	75.73	23:20	FOS (70% raftilose, 30% raftiline) 2 sachets daily	PPSV 23 H1N1: Caledonia A: Moscow (subtype AC3N2), Sydney B: Belgium (code 184-93)	Serum Ig/sIgA/Ab titers/cytokine secretion/lymphocyte proliferation/episode of URI	3
Langkamp-Henken 2004 [38]	USA (26 weeks)	66 healthy elders (47%:53%)	81.54	34:32	High oleic safflower oil, soybean oil, FOS, structured TG 8 oz. daily	H1N1: Beijing/262/95 H3N2: Sydney/5/97 B: Yamanashi/166/98 (B/Beijing/184/93)	Ab titers/lymphocyte proliferation/daily symptoms of URI	NR
Langkamp-Henken 2006 [37]	USA (10 weeks)	157 frail elders in LTC facilities (28%:72%)	83.36	76:72	Antioxidants, B vitamins, selenium, zinc, FOS, structured TG 240 mL daily	H1N1: Caledonia/20/99 H3N2: Panama/2007/99 B: Hong Kong/1434/2002	Cytokine studies/lymphocyte activation markers/immune cell phenotypes	NR
Nagafuchi 2015 [24]	Japan (14 weeks)	24 enteral tube feeding hospitalized elders (46%:54%)	80.30	12:12	BGS (1.65 µg/100 kcal), DHNA, GOS (0.4 g/100 kcal), fermented milk products	H1N1: California/7/2009 H3N2: Victoria/210/2009 B: Brisbane/60/2008	Ab titers/blood biochemical indices/intestinal bacterial populations	Nil
Lomax 2015 [25]	UK (8 weeks)	49 healthy adults (26%:74%)	54.98	22:21	50:50 mixture of long-chain inulin and oligofructose 8 g daily	H1N1: Brisbane/59/2007 H3N2: Brisbane/10/2007 B: Florida/4/2006	HI titers/total plasma Ig/vaccine-specific Ig/NK cell activity, immune cell phenotypes bactericidal activity, T-cell activity	NR
Akatsu 2016 [23]	Japan (8 weeks)	23 PEG-fed bedridden hospitalizedelders (13%:87%)	81.00	12:11	Heat-treated lactic acid bacteria-fermented milk products, GOS 4 g/day, BGS 0.4 g/day	H1N1: Solomon Islands/3/2006 H3N2: Hiroshima/52/2005 B: Malaysia/2506/2004	Hi titers/cytokine levels/biochemical markers	NR

*L. fermentum*: *Lactobacillus fermentum*; *L. casei*: *Lactobacillus casei*; *L. plantarum*: *Lactobacillus plantarum*; *L. paracasei*: *Lactobacillus paracasei*; *L.GG*: *Lactobacillus GG*; *B. longum*: *Bifidobacterium longum*; CFU: colony-forming unit; LTC: long term care facilities; FOS: fructo-oligosaccharides; GOS: galacto-oligosaccharide; TG: triglycerol; BGS: bifidogenic growth stimulator; DHNA: 1,4-dihydroxy-2-naphthoic acid; PPSV 23: pneumococcal polysaccharide vaccine 23; PEG: percutaneous endoscopic gastrostomy; Ig: immunoglobulin; sIg: specific Immunoglobulin, Ab: antibody, PBMC: peripheral blood mononuclear cells, GI-OS: gluco-oligosaccharide, AEs: adverse events, CMV: cytomegalovirus, NK cell: nature killer cell, NR: not-regulated, Tx: treatment, URI: upper respiratory tract infection, Nil: none.

**Table 2:** Subgroup analysis of odds ratio of seroprotection and seroconversion rate for different influenza strains based on health status of participants.

Subgroup	H1N1	H3N2	B
**Health elders**			
Seroprotection	2.46 (1.15–5.26) †	2.27 (0.94–5.47)	1.19 (0.56–2.50)
Seroconversion	2.93 (1.47–5.87) *	3.68 (1.11–12.25) †	2.69 (1.51–4.78) *
**Health young/middle-aged adults**			
Seroprotection	0.85 (0.32–2.25)	4.20 (1.34–13.16) †	1.22 (0.48–3.12)
Seroconversion	0.62 (0.18–2.14)	3.46 (1.22–9.83) †	1.08 (0.40–2.88)
**Hospitalized elders**			
Seroprotection	2.06 (1.11–3.82) †	2.83 (0.97–8.21)	0.80 (0.43–1.49)
Seroconversion	0.98 (0.51–1.89)	0.61 (0.32–1.18)	2.05 (0.92–4.58)

* *p* < 0.005, † *p* < 0.05.

**Table 3:** Subgroup analysis of odds ratio of seroprotection and seroconversion rate for different influenza vaccine strains based on supplements.

Subgroup	H1N1	H3N2	B
Probiotics			
Seroprotection	1.73 (0.79–3.80)	2.68 (1.25–5.72) †	1.23 (0.65–2.33)
Seroconversion	1.91 (0.68–5.38)	3.52 (1.45–8.53) *	2.24 (1.24–4.06) *
Prebiotics			
Seroprotection	1.88 (1.06–3.33) †	3.11 (1.25–7.71) †	0.84 (0.48–1.48)
Seroconversion	0.99 (0.54–1.83)	1.31 (0.22–7.98)	1.78 (0.87–3.63)

* *p* < 0.01, † *p* < 0.05.

## Supplementary Materials

The following are available online at www.mdpi.com/2072-6643/9/11/1175/s1, Table S1: PRISMA checklist, Table S2: Detailed searching strategy, Table S3: Quality assessment of included studies based on Cochrane risk of Bias tool, Table S4: Publication bias assessment with Egger’s regression, Figure S1: Funnel plot of seroprotection rate of H1N1 strain, Figure S2: Funnel plot H3N2 seroprotection, Figure S3: Funnel plot B seroprotection, Figure S4: Funnel plot H1N1 seroconversion, Figure S5: Funnel plot H3N2 seroconversion, Figure S6 :Funnel plot B seroconversion.
