# Supplementary material for: Effect of Probiotics and Prebiotics on Immune Response to Influenza Vaccination in Adults: A Systematic Review and Meta-Analysis of Randomized Controlled Trials
Source: Nutrients. 2017 Oct 27;9(11):1175. doi: 10.3390/nu9111175 (PMC5707647; doi:10.3390/nu9111175)
Supplement: Supplementary file 1 [file nutrients-09-01175-s001.zip › nutrients-229717-supplementary/Table S2 Detailed searching strategy.docx]

Table S2. Searching strategy

PubMed

| ((((Flu Vaccine* OR Afluria OR Influenza Vaccine* OR Afluria OR Influenzavirus Vaccine* OR LAIV vaccine OR FluMist OR CAIV-T vaccine OR Trivalent Live Attenuated Influenza Vaccine OR Influenza Virus Vaccine*)) OR ((((Influenza, Human) OR (Influenza* OR flu)))  in All Fields |
| --- |
| AND |
| ((vaccination) OR vaccine*))))  in All Fields |
| AND |
| ((((((((Probiotics) OR Bifidobacterium longum) OR Lactobacillus rhamnosus) OR (Lactic acid bacteria OR Lactobacillus acidophilus OR Lactobacillus amylovorus OR Lactobacillus Streptococcus faecalis OR L. acidophilus OR B. lactis OR Bifidobacterium OR B. bifidum OR B. longum OR Bifidobacter* OR Lactobacillus casei OR Lactobacillus paracasei OR Lactobacillus rhamnosus OR Lactobacillus GG OR Culturelle)) OR probiotic*)) OR ((Prebiotics) OR ((Prebiotic* OR Oligosaccharid*)))) OR ((Synbiotics) OR Synbiotic*)  in All Fields |

Embase

| Influenza Vaccines OR Flu Vaccine* OR Afluria OR Influenza Vaccines OR Flu Vaccine* OR Afluria OR Influenzavirus Vaccine* OR LAIV vaccine OR FluMist OR CAIV-T vaccine OR Trivalent Live Attenuated Influenza Vaccine OR Influenza Virus Vaccine* |
| --- |
| AND |
| vaccination OR vaccine* |
| AND |
| ((((((((Probiotics) OR Bifidobacterium longum) OR Lactobacillus rhamnosus) OR (Lactic acid bacteria OR Lactobacillus acidophilus OR Lactobacillus amylovorus OR Lactobacillus Streptococcus faecalis OR L. acidophilus OR B. lactis OR Bifidobacterium OR B. bifidum OR B. longum OR Bifidobacter* OR Lactobacillus casei OR Lactobacillus paracasei OR Lactobacillus rhamnosus OR Lactobacillus GG OR Culturelle)) OR probiotic*)) OR ((Prebiotics) OR ((Prebiotic* OR Oligosaccharid*)))) OR ((Synbiotics) OR Synbiotic*)) |

Cochrane

| Influenza Vaccines OR Flu Vaccine* OR Afluria OR Influenza Vaccines OR Flu Vaccine* OR Afluria OR Influenzavirus Vaccine* OR LAIV vaccine OR FluMist OR CAIV-T vaccine OR Trivalent Live Attenuated Influenza Vaccine OR Influenza Virus Vaccine* |
| --- |
| AND |
| vaccination OR vaccine* |
| AND |
| ((((((((Probiotics) OR Bifidobacterium longum) OR Lactobacillus rhamnosus) OR (Lactic acid bacteria OR Lactobacillus acidophilus OR Lactobacillus amylovorus OR Lactobacillus Streptococcus faecalis OR L. acidophilus OR B. lactis OR Bifidobacterium OR B. bifidum OR B. longum OR Bifidobacter* OR Lactobacillus casei OR Lactobacillus paracasei OR Lactobacillus rhamnosus OR Lactobacillus GG OR Culturelle)) OR probiotic*)) OR ((Prebiotics) OR ((Prebiotic* OR Oligosaccharid*)))) OR ((Synbiotics) OR Synbiotic*)) |

CINAHL

| ((((Flu Vaccine* OR Afluria OR Influenza Vaccine* OR Afluria OR Influenzavirus Vaccine* OR LAIV vaccine OR FluMist OR CAIV-T vaccine OR Trivalent Live Attenuated Influenza Vaccine OR Influenza Virus Vaccine*)) OR ((((Influenza, Human) OR (Influenza* OR flu))) |
| --- |
| AND |
| ((vaccination) OR vaccine*)))) |
| AND |
| ((((((((Probiotics) OR Bifidobacterium longum) OR Lactobacillus rhamnosus) OR (Lactic acid bacteria OR Lactobacillus acidophilus OR Lactobacillus amylovorus OR Lactobacillus Streptococcus faecalis OR L. acidophilus OR B. lactis OR Bifidobacterium OR B. bifidum OR B. longum OR Bifidobacter* OR Lactobacillus casei OR Lactobacillus paracasei OR Lactobacillus rhamnosus OR Lactobacillus GG OR Culturelle)) OR probiotic*)) OR ((Prebiotics) OR ((Prebiotic* OR Oligosaccharid*)))) OR ((Synbiotics) OR Synbiotic*) |

Airiti

| 流感 OR 流行性感冒  OR 感冒 |
| --- |
| AND |
| 疫苗 |
| AND |
| 益生菌 OR 乳酸菌 OR 龍根菌 OR益菌生 OR 益生源 OR 合生元 OR 共生質 OR 合益菌 |

NTLTD

| 流感 + 流行性感冒 + 感冒 |
| --- |
| AND |
| 疫苗 |
| AND |
| 益生菌 + 乳酸菌 + 龍根菌 + 益菌生 + 益生源 + 合生元 + 共生質 + 合益菌 |
