# Supplementary material for: Effect of Probiotics and Prebiotics on Immune Response to Influenza Vaccination in Adults: A Systematic Review and Meta-Analysis of Randomized Controlled Trials
Source: Nutrients. 2017 Oct 27;9(11):1175. doi: 10.3390/nu9111175 (PMC5707647; doi:10.3390/nu9111175)
Supplement: Supplementary file 1 [file nutrients-09-01175-s001.zip › nutrients-229717-supplementary/Table S3 Quality assessment of included studies based on Cochrane risk of Bias tool.docx]

Table S3. Risk of bias assessment of each included study ^a^

| Study Validity  Domains | Sequence generation | Allocation Concealment | Blinding of participants and personnel and outcome assessors | Incomplete outcome data | Selective outcome reporting | Other sources  of bias |
| --- | --- | --- | --- | --- | --- | --- |
| **Probiotics** |  |  |  |  |  |  |
| Olivares 2007 | Low | Unclear^b^ | Unclear^b^ | Low | Low | Low |
| French & Penny 2009 | Low | Low | Low | Low | Low | Uncertain^f^ |
| Boge 2009 (pilot) | Low | Low | Low | High^d^ | Low | Uncertain^f^ |
| Boge 2009 (confirmed) | Low | Low | Low | High^d^ | Low | Uncertain^f^ |
| Namba 2010 | Low | Unclear^b^ | Unclear^b^ | High^d^ | Low | Uncertain^f^ |
| Davidson 2011 | Low | Low | Low | Low | Low | Low |
| Van Puyenbroeck 2012 | Low | Low | Low | High^d^ | High^e^ | Uncertain^f^ |
| Rizzardini 2012 | Low | Low | Low | Low | Low | Uncertain^f^ |
| Bosch 2012 | Unclear^b^ | Unclear^b^ | Low | High^d^ | Low | High^g^ |
| Akatsu 2013a (letter) | Unclear^b^ | Unclear^b^ | High^C^ | Low | Uncertain^b^ | Uncertain^f^ |
| Akatsu 2013b (paper) | Low | Unclear^b^ | Unclear^b^ | Low | Low | Uncertain^f^ |
| Jespersen 2015 | Low | Low | Low | Low | High^e^ | Uncertain^f^ |
| Maruyama 2016 | Low | Low | Low | Low | Low | Low |

Table S3. Risk of bias assessment of each included study (con’t)

| Study Validity  Domains | Sequence generation | Allocation Concealment | Blinding of participants and personnel and outcome assessors | Incomplete outcome data | Selective outcome reporting | Other sources  of bias |
| --- | --- | --- | --- | --- | --- | --- |
| **Prebiotics** |  |  |  |  |  |  |
| Bunout 2002 | Low | Unclear^b^ | Low | High^d^ | Low | Uncertain^f^ |
| Langkamp-Henken  2004 | Low | Low | Unclear^b^ | High^d^ | Low | Uncertain^f^ |
| Langkamp-Henken  2006 | Unclear^b^ | Low | Low | High^d^ | Low | High^g^ |
| Nagafuchi 2015 | Unclear^b^ | Unclear^b^ | High^c^ | Low | Low | Uncertain^f^ |
| Lomax 2015 | Unclear^b^ | Low | Unclear^b^ | High^d^ | High^e^ | Uncertain^f^ |
| Akatsu 2016 | Unclear^b^ | Unclear^b^ | High^c^ | Low | Low | Uncertain^f^ |
| **Synbiotics** |  |  |  |  |  |  |
| Enami 2017 | Unclear^b^ | Low | Low | High^d^ | Unclear | Unclear^f^ |

a. Each domain has been evaluated as being “High”, “Low”, or “Unclear” regarding the risk of bias following the guidelines of Cochrane Collaboration’s tool for assessing risk of bias “Low” in all Domains would place a study at “Low Risk of Bias”; “High” in any of the Domains would place a study at “High Risk of Bias”; “Unclear” in any of the domains would place the study at “Unclear Risk of Bias”

b. Not mentioned

c. Un-blinded, open-labeled

d. Drop-off rate > 10%

e. Missing data / data lost

f. Conflict of interest, financial supports

g. Authors employed by funding companies
