# Supplementary material for: Effect of Probiotics and Prebiotics on Immune Response to Influenza Vaccination in Adults: A Systematic Review and Meta-Analysis of Randomized Controlled Trials
Source: Nutrients. 2017 Oct 27;9(11):1175. doi: 10.3390/nu9111175 (PMC5707647; doi:10.3390/nu9111175)
Supplement: Supplementary file 1 [file nutrients-09-01175-s001.zip › nutrients-229717-supplementary/Table S4 Publication bias assessment with Egger's regression.docx]

**Table S4** Publication bias assessment using Egger’s regression for different influenza vaccine strains

| **Subgroup** | **H1N1** | **H3N2** | **B** |
| --- | --- | --- | --- |
| ***P* value** |  |  |  |
| Seroprotection | 0.37 | 0.86 | 0.61 |
| Seroconversion | 0.95 | 0.76 | 0.47 |
