# Supplementary material for: Effect of Probiotics and Prebiotics on Immune Response to Influenza Vaccination in Adults: A Systematic Review and Meta-Analysis of Randomized Controlled Trials
Source: Nutrients. 2017 Oct 27;9(11):1175. doi: 10.3390/nu9111175 (PMC5707647; doi:10.3390/nu9111175)
Supplement: Supplementary file 1 [file nutrients-09-01175-s001.zip › nutrients-229717-supplementary/Table S5. Compliance of supplements in different studies.docx]

Table S5. Compliance of supplements in different studies

| Study | Definition/measurement of compliance | Results (in intervention group) | Used in meta-analysis |
| --- | --- | --- | --- |
| Enani 2017 | Counting returned sachets and by copy numbers of *B. longum*, assessed by qPCR | Data not shown | No |
| Maruyama 2016 | Not mentioned | Good compliance (98.8+/-3.0%) | No |
| Lomax 2015 | Compliance were assessed by returning unused sachets and Synergy1 increased fecal bifidobacteria numbers | Good compliance (100%) | Yes, prebiotics |
| Jesperson 2015 | Counting the number of returned unopened bottles during the intervention period | Good compliance (99.9%) | No |
| Van Puyenboreck 2012 | Compliance in consumption of the test product was also checked on the basis of the diary records | Good compliance (no data) | No |
| Rizzardini 2012 | Assessment of compliance was based on subjects’ recordings of missing doses | Good compliance (99.1%/98.2%) | No |
| Boge 2009 (pilot) | Compliance was monitored and recorded by the investigators at each visit. | Good compliance (97.0%) | Yes, probiotics |
| Boge 2009 (confirmed) | Compliance was monitored and recorded by the investigators at each visit. | Good compliance (96.3%) | Yes, probiotics |
| Olivares 2007 | By fecal detection of the probiotic strain | Data not shown | No |
| Langkamp-Henken 2006 | Measuring serum alpha-tocopherol and beta-carotene concentrations  (> 180mL was defined as evaluable subjects) | Not good compliance (56%)  (< 180mL was the main reason for noncompliance) | Yes, prebiotics |
| Langkamp-Henken 2004 | Measuring serum alpha-tocopherol concentration | Good compliance (no data) | No |
| Bunout 2002 | Not mentioned | Good compliance (95%) | No |
