# Supplementary material for: Effect of Probiotics and Prebiotics on Immune Response to Influenza Vaccination in Adults: A Systematic Review and Meta-Analysis of Randomized Controlled Trials
Source: Nutrients. 2017 Oct 27;9(11):1175. doi: 10.3390/nu9111175 (PMC5707647; doi:10.3390/nu9111175)
Supplement: Supplementary file 1 [file nutrients-09-01175-s001.zip › nutrients-229717-supplementary/Table S6. Subgroup analysis of odds ratio of seroprotection and seroconversion rate for difference vaccine strains based on different probiotic strains.docx]

Table S5. Subgroup analysis of odds ratio of seroprotection and seroconversion rate for difference vaccine strains based on different probiotic strains

| **Subgroup** | **H1N1** | **H3N2** | **B** |
| --- | --- | --- | --- |
| **Non-LGG** |  |  |  |
| Seroprotection | 2.46 (1.15 - 5.26)† | 2.27 (0.94 - 5.47) | 1.19 (0.56 - 2.50) |
| Seroconversion | 2.93 (1.49 - 5.78)* | 4.00 (2.49 - 6.43)* | 2.69 (1.51 - 4.78)* |
| **LGG** |  |  |  |
| Seroprotection | 0.73 (0.21 - 2.57) | 4.36 (0.96 - 19.86) | 1.36 (0.39 - 4.79) |
| Seroconversion | 0.35 (0.06 - 2.10) | 3.18 (0.86 - 11.79) | 1.09 (0.30 - 3.91) |

**p*<0.01, †*p*<0.05
