# Supplementary figures and images for: Effect of Probiotics and Prebiotics on Immune Response to Influenza Vaccination in Adults: A Systematic Review and Meta-Analysis of Randomized Controlled Trials
Source: Nutrients. 2017 Oct 27;9(11):1175. doi: 10.3390/nu9111175 (PMC5707647; doi:10.3390/nu9111175)

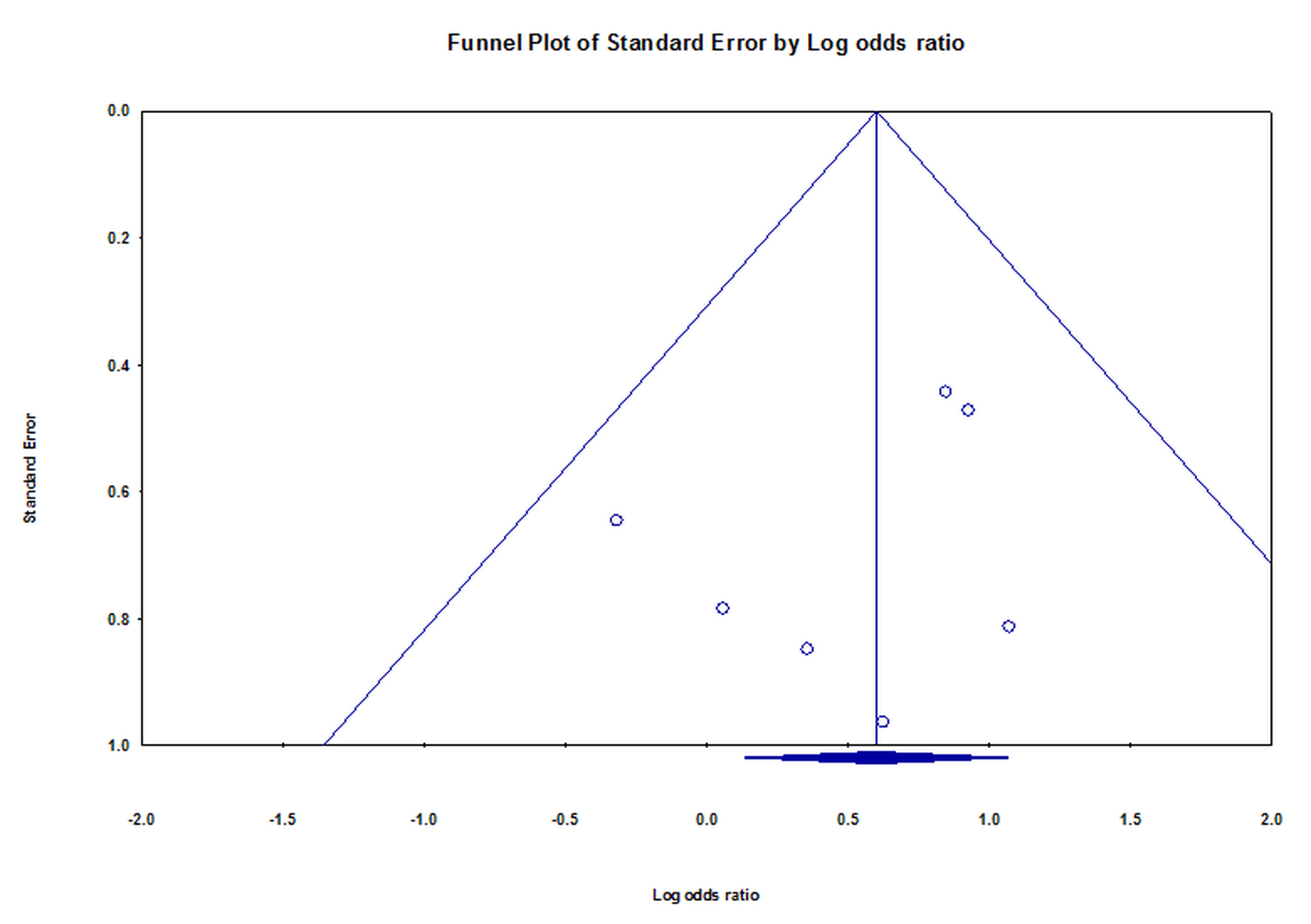

Supplement: Supplementary file 1 [file nutrients-09-01175-s001.zip › nutrients-229717-supplementary/Figure S1 Funnel plot H1N1 seroprotection.png]

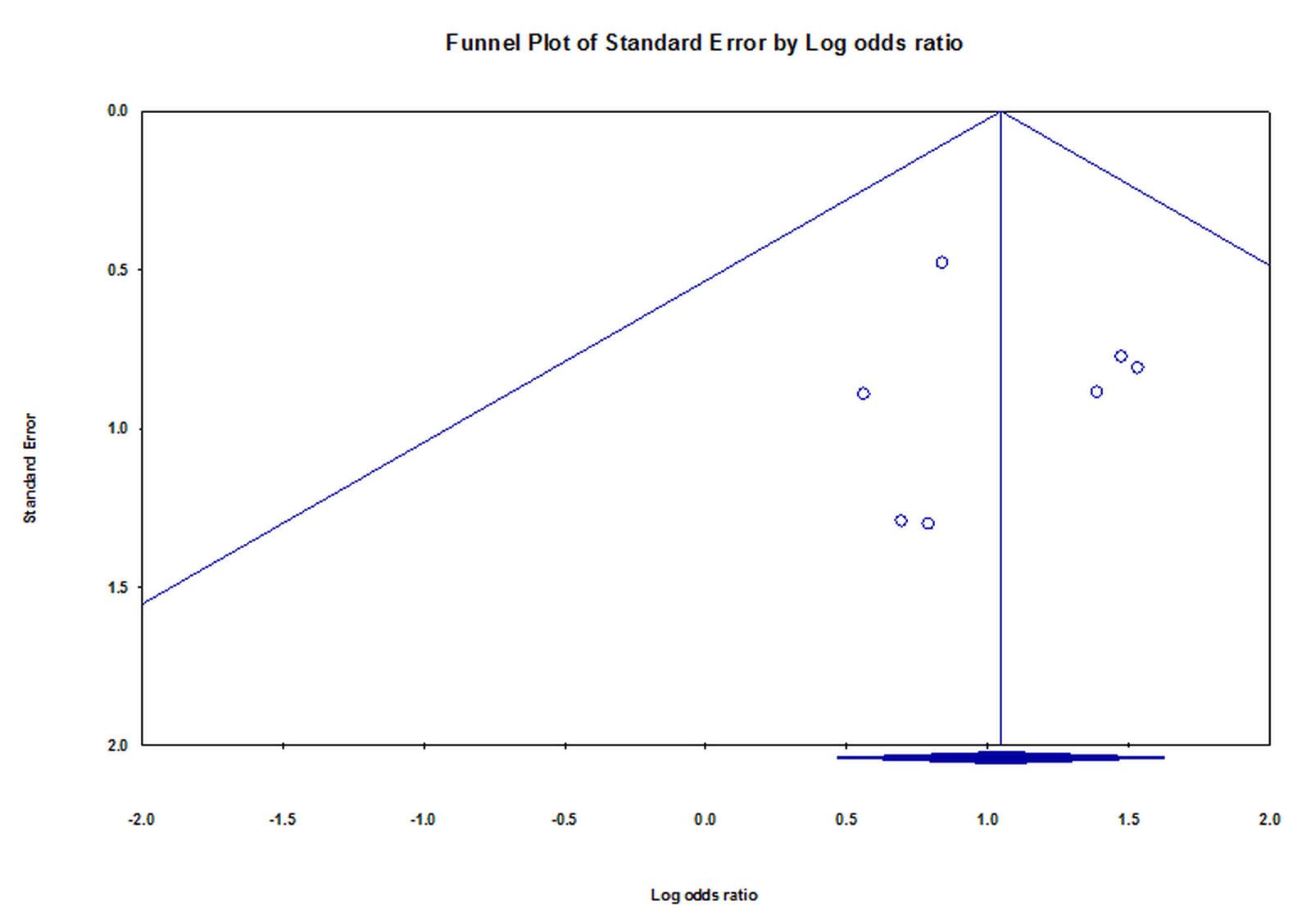

Supplement: Supplementary file 1 [file nutrients-09-01175-s001.zip › nutrients-229717-supplementary/Figure S2 Funnel plot H3N2 seroprotection.png]

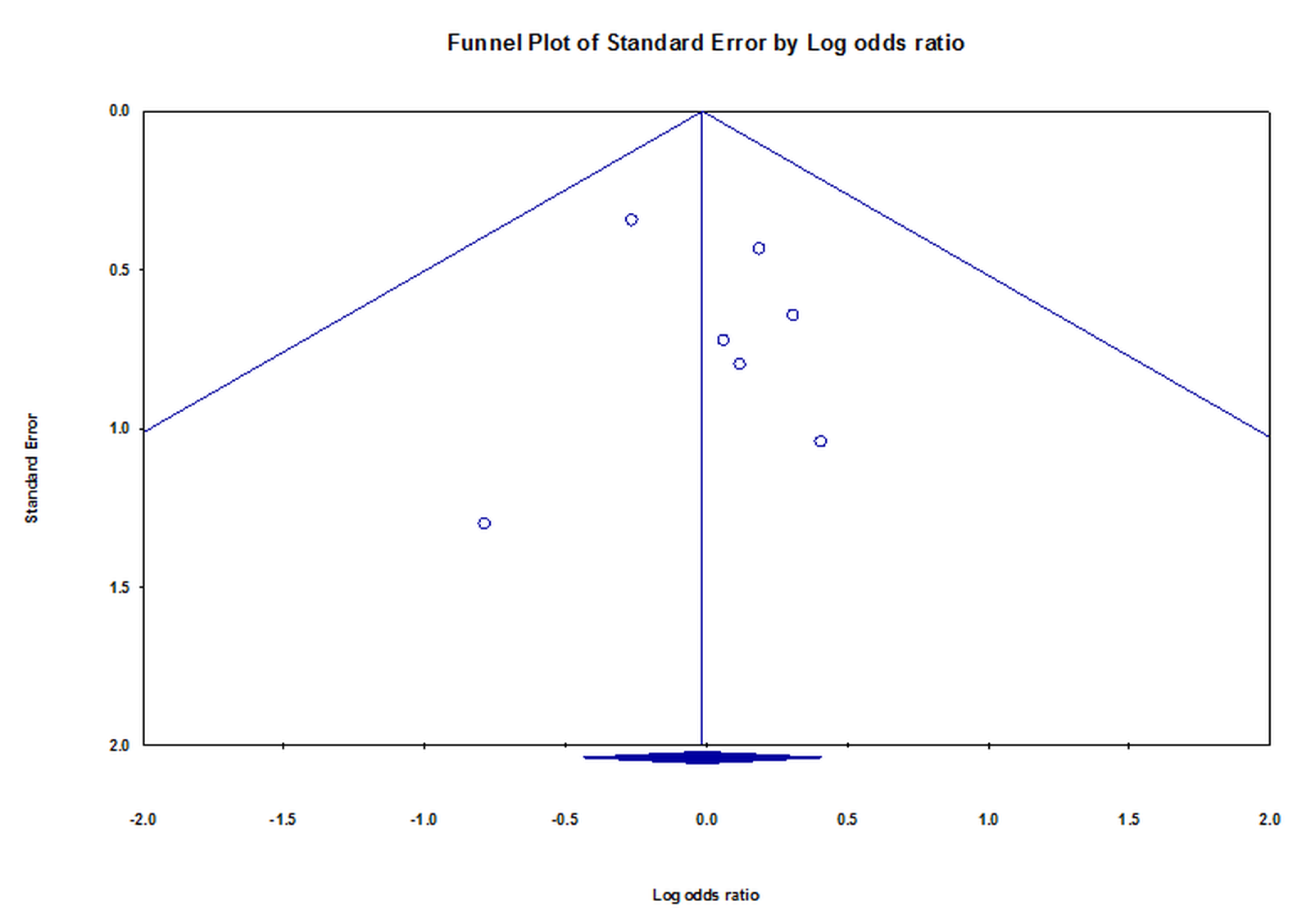

Supplement: Supplementary file 1 [file nutrients-09-01175-s001.zip › nutrients-229717-supplementary/Figure S3 Funnel plot B seroprotection.png]

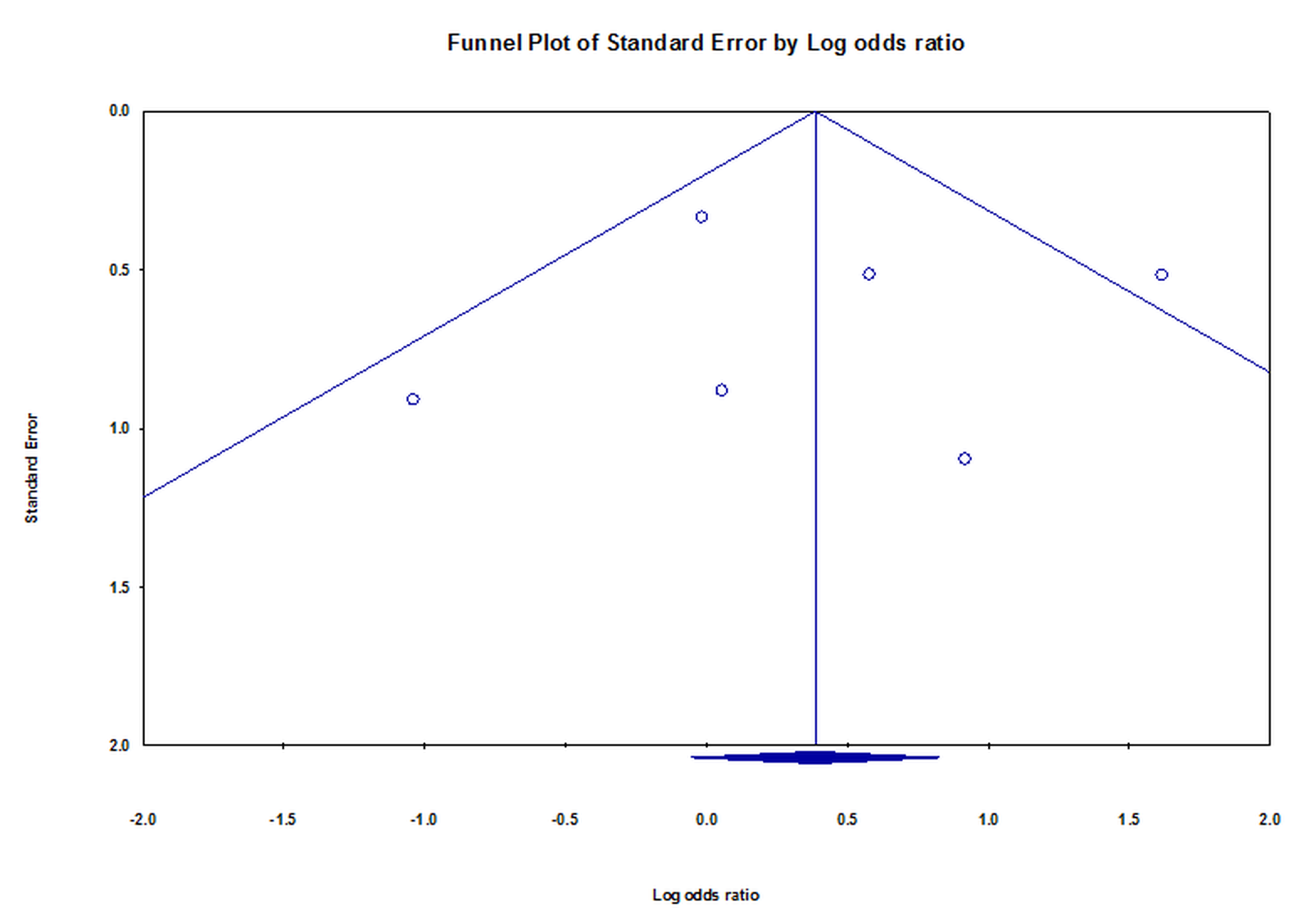

Supplement: Supplementary file 1 [file nutrients-09-01175-s001.zip › nutrients-229717-supplementary/Figure S4 Funnel plot H1N1 seroconversion.png]

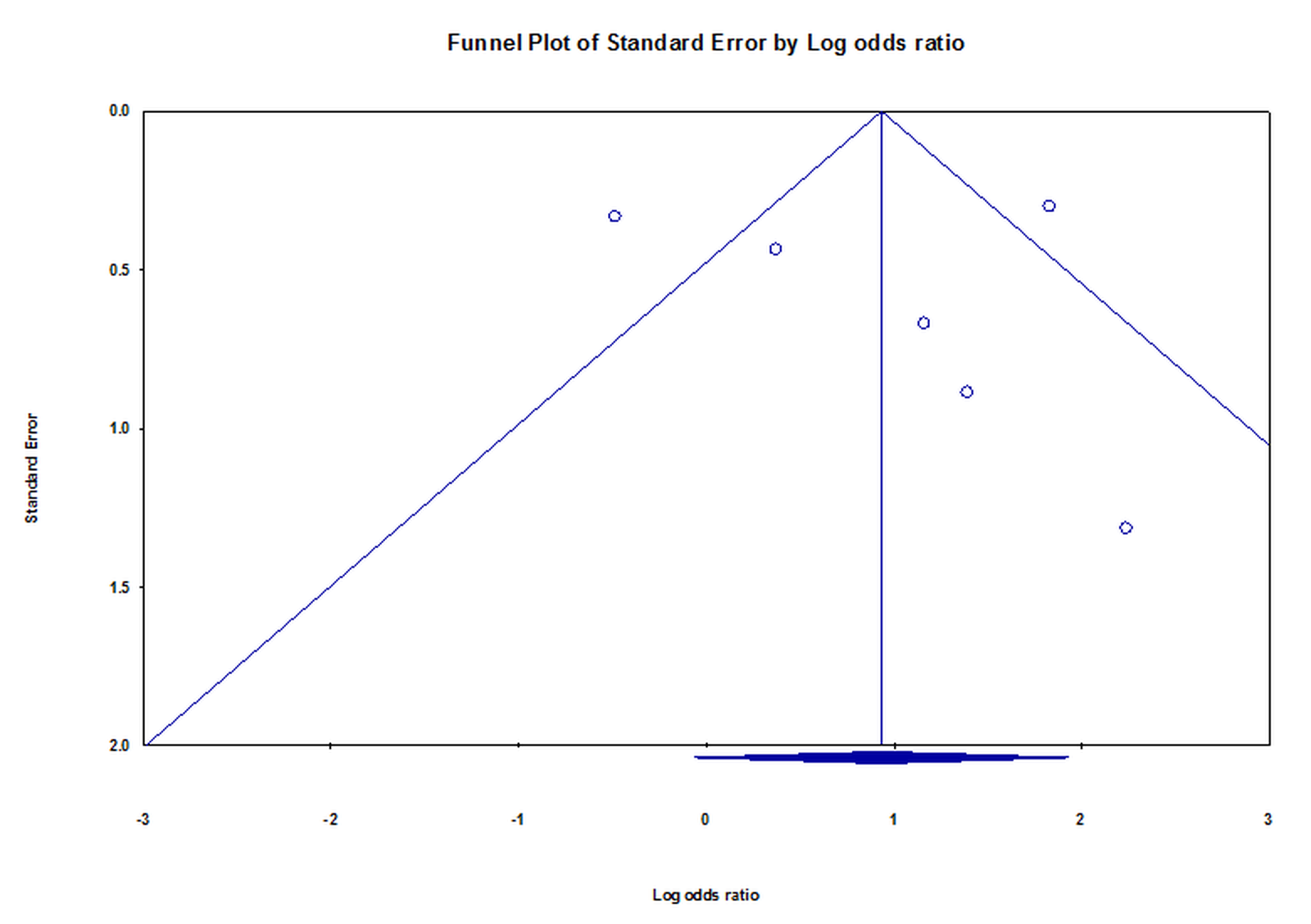

Supplement: Supplementary file 1 [file nutrients-09-01175-s001.zip › nutrients-229717-supplementary/Figure S5 Funnel plot H3N2 seroconversion.png]

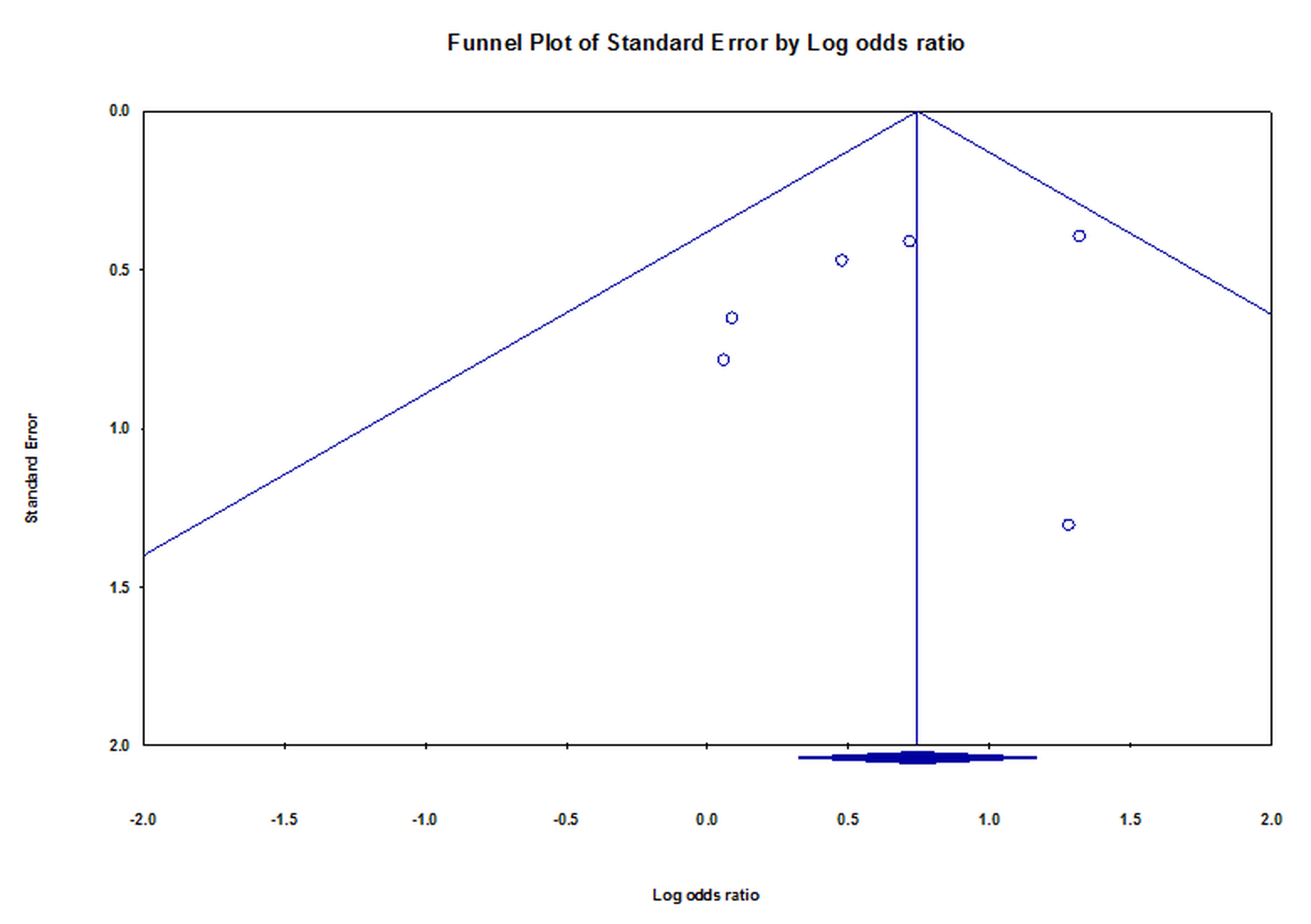

Supplement: Supplementary file 1 [file nutrients-09-01175-s001.zip › nutrients-229717-supplementary/Figure S6 Funnel plot B seroconversion.png]
